# Supplementary material for: Chromothripsis during telomere crisis is independent of NHEJ, and consistent with a replicative origin
Source: Genome Res. 2019 May;29(5):737–49. doi: 10.1101/gr.240705.118 (PMC6499312; doi:10.1101/gr.240705.118)
Supplement: Supplemental Material [file supp_gr.240705.118_Supplemental_file_1.zip › contigs/annotated_contigs/DB50/contig.2.DB50_length_686_mean_cov_10.7113702624.docx]

**DB50_length_686_mean_cov_10.7113702624**

```
ACTGTTGGAGCAGAATTTTTTACCCTGGATCAATTGTGTGCCTTCCTGATGATACTTTAATAGAACAAGTGGGCACAGAGTGACAGATG
 >chr12:47809038-47809397 - E=3e-204
ATGACGGGAGGCTCAGACACAAAGTAGAACTACTGTTGCCTGGAGCCAAACAAAAATTATACCATTTAAAAACTCAGAATCCAAGAGCT

AATATGTTGTCCACAAAAGAACAAAAGGCAGAAAGTTTATGCTAAAATGAGGTAAGTTATAAATGTCAGTAACTCATAAAATGGGTATG

TCCCATTGGGACCTAAAGAGTTAATATATAAAAGGAAACAGATTGAGGTCAGGTAGCCTCATGAAAATCTCAAATGCTGTGACACAGAG

ACA|CCGACAACAGCAAAGCAAACCCAAACAGAACCCTCTCAACCTAAACAATCTTCCTTTCCCCTTTTAACTCTGAAGCTCTTATAAA
     >chr12:47808116-47808444 - E=9e-181
AAACAGATATTTCAAGGATGTCCTTTAAGCTAAATACAAACTAGAACATTGAGGTAAAACACAATTATCTGCCCTAAAGTAAAAATATA

GATTTTTAATATTTCCAATAAATATATTTCAAATCACCAGTTTGAGGTGTGGAATAAGTTCATATTTATTTTAGTGTTGAAACCATAGT

AAGTGGAAAAAAAAACCCTCTAGGTTGACAAGAAGACCAATCTTTCTCCTAGGATTTCCCTCCC
```
